# Disparities in kidney care in vulnerable populations: A multinational study from the ISN-GKHA

**DOI:** 10.1371/journal.pgph.0004086

**Published:** 2024-12-20

**Authors:** Robin L. Erickson, Nivedita Kamath, Arpana Iyengar, Adebowale Ademola, Christopher Esezobor, Rowena Lalji, Erin Hedin, Silvia Arruebo, Fergus J. Caskey, Sandrine Damster, Jo-Ann Donner, Vivekanand Jha, Adeera Levin, Masaomi Nangaku, Syed Saad, Marcello Tonelli, Feng Ye, Ikechi G. Okpechi, Aminu K. Bello, David W. Johnson

**Affiliations:** 1 Paediatric Kidney Service, Starship Children’s Hospital, University of Auckland, Auckland, New Zealand; 2 Department of Pediatric Nephrology, St. John’s Medical College Hospital, Bangalore, India; 3 Department of Paediatric Nephrology, St.John’s National Academy of Health Sciences, Bangalore, India; 4 Department of Paediatrics, Faculty of Clinical Sciences, College of Medicine, University of Ibadan / University College Hospital Ibadan, Ibadan, Oyo State, Nigeria; 5 Department of Paediatrics, Faculty of Clinical Sciences, College of Medicine, University of Lagos, Lagos, Nigeria; 6 Centre for Kidney Disease Research, University of Queensland, Brisbane, Queensland, Australia; 7 Department of Nephrology, Queensland Children’s Hospital, Brisbane, Queensland, Australia; 8 Metro South and Integrated Nephrology and Transplant Services (MINTS), Princess Alexandra Hospital, Brisbane, Queensland, Australia; 9 Division of Pediatric Nephrology, Department of Pediatrics, University of Alberta, Edmonton, Alberta, Canada; 10 The International Society of Nephrology, Brussels, Belgium; 11 Population Health Sciences, Bristol Medical School, University of Bristol, Bristol, United Kingdom; 12 George Institute for Global Health, University of New South Wales (UNSW), New Delhi, India; 13 School of Public Health, Imperial College, London, United Kingdom; 14 Manipal Academy of Higher Education, Manipal, India; 15 Division of Nephrology, Department of Medicine, University of British Columbia, Vancouver, British Columbia, Canada; 16 Division of Nephrology and Endocrinology, The University of Tokyo Graduate School of Medicine, Tokyo, Japan; 17 Division of Nephrology and Immunology, Faculty of Medicine and Dentistry, University of Alberta, Edmonton, Alberta, Canada; 18 Department of Medicine, University of Calgary, Calgary, Alberta, Canada; 19 Canada and Pan-American Health Organization/World Health Organization’s Collaborating Centre in Prevention and Control of Chronic Kidney Disease, University of Calgary, Calgary, Alberta, Canada; 20 Division of Nephrology and Hypertension, University of Cape Town, Cape Town, South Africa; 21 Kidney and Hypertension Research Unit, University of Cape Town, Cape Town, South Africa; 22 Department of Kidney and Transplant Services, Princess Alexandra Hospital, Brisbane, Queensland, Australia; 23 Translational Research Institute, Brisbane, Queensland, Australia; 24 Australasian Kidney Trials Network at the University of Queensland, Brisbane, Queensland, Australia; Nanyang Technological University, SINGAPORE

## Abstract

Vulnerable populations, such as the elderly, children, displaced people, and refugees, often encounter challenges in accessing healthcare. In this study, we used data from the third iteration of the International Society of Nephrology Global Kidney Health Atlas (ISN-GKHA) to describe kidney care access and delivery to vulnerable populations across countries and regions. Using data from an international survey of clinicians, policymakers, and patient advocates, we assessed the funding and coverage of vulnerable populations on all aspects of kidney replacement therapies (KRT). Overall, 167 countries or jurisdictions participated in the survey, representing 97.4% of the world’s population. Children had less access than adults to KRT: hemodialysis (HD) in 74% of countries, peritoneal dialysis (PD) in 53% of countries, and kidney transplantation (KT) in 80% of countries. Available nephrologist workforce for pediatric kidney care was much lower than for adults (0.69 per million population [pmp] vs 10.08 pmp). Refugees or displaced people with kidney failure did not have access to HD, PD, or KT in 21%, 33%, and 37% of the participating countries, respectively. Low-income countries (LICs) were less likely to provide KRT access to refugees compared to high-income countries (HICs): HD: 13% vs 22%; PD: 19% vs 61%; KT: 30% vs 44%. Testing for kidney disease was routinely offered to elderly people in only 61% of countries: LICs (45%), lower-middle-income countries (56%), upper-middle-income countries (54%), and HICs (75%). Equitable access to kidney care for vulnerable people, particularly for children and displaced people, remains an area of unmet need. Strategies are needed to address this issue.

## Introduction

Universal health coverage (UHC) allows for all individuals to receive the quality health services that they need, when and where they need it, and is one of the United Nations Sustainable Development Goals [1]. Central to achieving this goal is the existence of health systems that are appropriately financed and structured to allow a skilled workforce with the medications and tools needed to provide equitable care to everyone, regardless of environmental, political, or social upheavals.

Kidney failure is a significant healthcare burden due to the high costs of care and its substantial impact on quality of life, including the burden of lifelong kidney replacement therapies (KRT; hemodialysis [HD], peritoneal dialysis [PD], and kidney transplantation [KT]). Vulnerable populations are at increased risk of developing kidney disease and also have several challenges accessing kidney care [2]. These populations may be vulnerable for several reasons, including long-standing or acute environmental factors, the presence of comorbidity, humanitarian emergencies or as a result of social determinants such as poverty, education, age, systemic discrimination, or political conflicts [3, 4].

The International Society of Nephrology Global Kidney Health Atlas (ISN-GKHA) is a multinational initiative that assesses the availability, accessibility, and affordability of kidney care using a framework of the World Health Organization UHC domains, including service delivery, health workforce, information systems, financing, leadership, and medicines and medical products. In this study, we used data from the ISN-GKHA [5] to report the availability and accessibility of KRT to vulnerable populations, defined as children, the elderly, those with housing insecurities, racial/ethnic minorities, displaced people, and refugees.

## Methods

The 2023 ISN-GKHA is an international project that describes the global and regional capacity to provide care to people with kidney disease (chronic kidney disease [CKD] and kidney failure). A full description of methodologies has been published [5]. In brief, two strategies were used to obtain country-level data on kidney care capacity: an in-depth literature review of published data, grey literature, kidney registries, and databases, and a multinational, cross-sectional, online survey of opinion leaders. For this study, only survey data were utilized.

### Survey data

The survey was developed and validated through a series of reviews with content experts, the ISN Executive Committee, and ISN regional leadership. After being peer-reviewed for content validity and comprehensiveness, the survey was piloted on the 10 ISN regional boards: Africa, Middle East, North America and the Caribbean, Latin America, North and East Asia, South Asia, Oceania and South East Asia (OSEA), Western Europe, Eastern and Central Europe, and the Newly Independent States (NIS) and Russia. This served to identify any logistical and feasibility matters. The survey was available in English, French, and Spanish and accompanied by a detailed information sheet about the ISN-GKHA, instructions for completion, and a glossary defining key terms used in the survey.

Three leaders were identified from each country to participate in the survey, including a nephrology society leader, a policymaker, and a leader of a patient representative organization. From June 1 to September 30, 2022, the key stakeholders were provided with a link to the survey in electronic format on REDCap (www.redcapcloud.com). Within country heterogeneity was queried of all respondents and identification of additional potential respondents was requested. Email and telephone follow-ups by ISN regional and national leaders during the survey window served to ensure complete and timely responses.

### Survey items

Certified translators collected and translated data from French and Spanish to English. Individual survey data were extracted and cleaned utilizing Microsoft Excel and then merged into a single global database file. This was stored in a secure, centralized computer system with automatic backup procedures. ISN regional boards reviewed responses from their regions to ensure responses were consistent and unambiguous; clarification was sought from responding stakeholders if concerns were present. A subset of questions from the whole ISN-GKHA survey were analyzed in this study (S1 Table). These survey questions assessed differences in availability of and access to all modalities of KRT between adults and children, availability of workforce for providing KRT in children, methods of identifying vulnerable populations such as refugees, availability of funding for KRT for vulnerable populations, availability of guidelines regarding measures to be taken for disaster preparedness, country representation on the ISN-Renal Disaster Relief Task Force (ISN-RDRTF), and availability of kidney disease testing for the elderly across countries.

### Data handling and statistical analysis

Analysis was conducted using STATA17 software (Stata Corporation, 2017). Responses were summarized using a descriptive statistical approach and reported as counts with percentages or medians with interquartile range (IQR). As each country was the unit of analysis, data from multiple respondents within the same country were synthesized into a single response. Results were stratified by ISN region and by World Bank income groups: low-income countries (LICs), lower-middle-income countries (LMICs), upper-middle-income countries (UMICs), and high-income countries (HICs) (estimated in June 2022).

### Ethics approval

Ethics approval was obtained from the University of Alberta Research Ethics Committee (protocol number: PRO00063121). Consent was obtained by email from survey respondents. Our study did not report experiments on humans and/or the use of human tissue samples.

## Results

### Survey response rate and participation

Of the 167 countries that participated in the ISN-GKHA, 162 responded to the vulnerable populations identification questions. The distribution of countries that participated included: high-income (HICs): n = 63; upper-middle income (UMICs): n = 37; lower-middle income (LMICs): n = 44; and low-income (LICs): n = 18.

### Identification of vulnerable populations

The healthcare systems in 70 (43%) countries had no mechanisms in place to identify vulnerable populations (specifically people with housing insecurity, racial or ethnic minorities, people living in poverty, and people affected by food insecurity). In four ISN regions, more than half of the countries did not have a system for identifying vulnerable populations: Africa (n = 20; 51%), Eastern and Central Europe (n = 10; 63%), the Middle East (n = 7; 64%), and South Asia (n = 5; 71%). The proportion of countries without a national system to identify vulnerable populations was highest in LICs (n = 11; 61%) and reduced with increasing country income levels (Table 1).

**Table 1:** Availability of systems for identifying vulnerable populations stratified by ISN region and World Bank income group (N, %).

	Vulnerable population identification system N (%)		
	Yes	No	Unknown
Overall	58 (36)	70 (43)	34 (21)
ISN region			
Africa	14 (36)	20 (51)	5 (13)
Eastern and Central Europe	3 (19)	10 (63)	3 (19)
Latin America	9 (43)	10 (48)	2 (10)
Middle East	3 (27)	7 (64)	1 (9)
NIS and Russia	4 (40)	3 (30)	3 (30)
North America and the Caribbean	5 (42)	4 (33)	3 (25)
North and East Asia	4 (67)	1 (17)	1 (17)
Oceania and South East Asia	5 (28)	8 (44)	5 (28)
South Asia	0 (0)	5 (71)	2 (29)
Western Europe	11 (50)	2 (9)	9 (41)
World Bank income group			
Low income	4 (22)	11 (61)	3 (17)
Lower-middle income	13 (30)	24 (55)	7 (16)
Upper-middle income	10 (27)	20 (54)	7 (19)
High income	31 (49)	15 (24)	17 (27)

Abbreviations: ISN–International Society of Nephrology; NIS–Newly Independent States

### Disparities in access to care for kidney failure and KRT between children and adults

**Fig 1:**
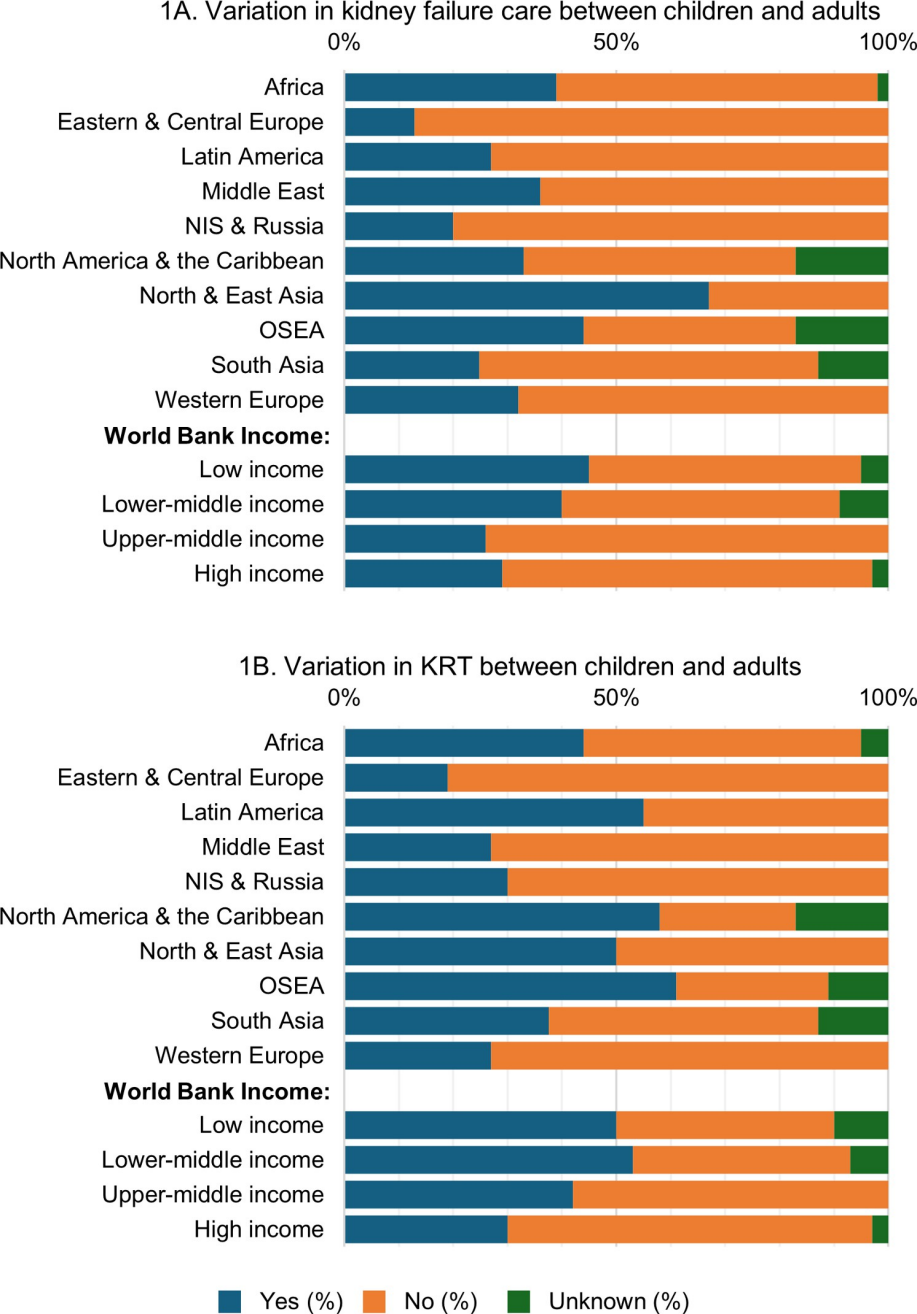
Variation in pediatric and adult care, by ISN region and World Bank income group.* **(A)** Variation in kidney failure care between children and adults; **(B)** Variation in KRT between children and adults. *Values represent the absolute number of countries in each category expressed as a percentage of total number of countries. Abbreviations: ISN–International Society of Nephrology; KRT–kidney replacement therapy; NIS–Newly Independent States; OSEA–Oceania and South East Asia.

Country respondents were asked to indicate whether there were differences between the end stage kidney disease (ESKD) care and KRT services available to children, compared to adults, in their respective countries and whether the access to these services were more or less available to children than adults, or not available at all to either group (S1 Table). Overall, variation in care delivery for kidney failure between children and adults was reported from 55 (33%) countries. This variation ranged from 13% (n = 2) in Eastern and Central Europe to 67% (n = 4) in North and East Asia. Variation was highest in LICs (n = 9; 45%) amongst country income categories (Fig 1A). Similarly, overall variation in access to KRT between children and adults was reported in 69 (42%) countries (Fig 1B). Across ISN regions, such variations ranged from 19% (n = 3) in Eastern and Central Europe to 61% (n = 11) in OSEA and were higher in LICs (n = 10; 50%) and LMICs (n = 24; 53%) than in other country income categories (Fig 1B).

**Fig 2:**
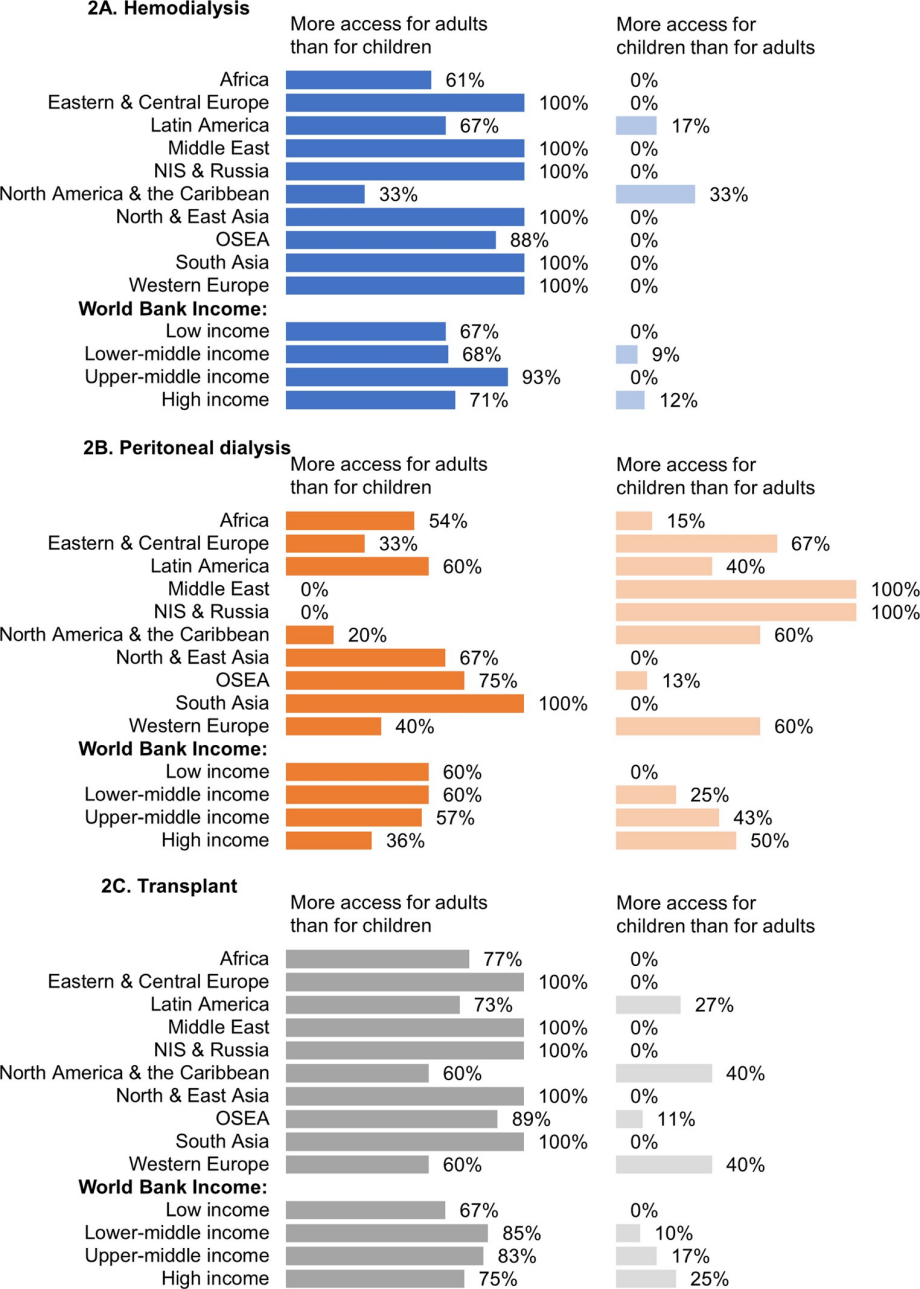
Variations in access to kidney replacement therapies between adults and children, by ISN region and World Bank income group.* **(A)** Variation in hemodialysis access; **(B)** Variation in peritoneal dialysis access; **(C)** Variation in kidney transplant access. *Values represent absolute number of countries in each category expressed as a percentage of total number of countries. Abbreviations: ISN–International Society of Nephrology; NIS–Newly Independent States; OSEA–Oceania and South East Asia.

Overall, HD was more accessible to adults than children in 46/62 (74%) countries and was available to adults while being unavailable to children in 12/62 (19%) countries. In Africa, 7 (39%) countries reported HD access to adults and not to children, while a third of countries in North America and the Caribbean (n = 2; 33%) had similar reporting (S2 Table). Across all ISN regions and country income groups, adults had more access to HD than children (Fig 2A). Similar to HD, in more than half of countries where PD was available, more adults than children had access to chronic PD (n = 28; 53%). In all countries in the Middle East and in NIS and Russia, children had more access to PD than adults, while in some countries in Africa (n = 2; 15%), North and East Asia (n = 1; 33%), and OSEA (n = 1; 13%), access to PD was available to children but not to adults (Fig 2B and S3 Table). Country income group variations in access to PD between adults and children were observed. Children had less access to KT than adults in 43/54 (80%) countries. A minority of countries in the ISN Latin America (n = 3; 27%), North America and the Caribbean (n = 2; 40%), OSEA (n = 1; 11%), and Western Europe (n = 2; 40%) regions reported that children had more access than adults (Fig 2C and S4 Table). Only countries in Africa (n = 3; 23%), representing 2 (33%) LICs and 1 (5%) LMIC, reported KT access to adults and not to children (S4 Table). Several barriers leading to disparities in care were identified from the qualitative responses provided in the survey (S5 Table). These included healthcare funding, access to KRT services, and the availability of pediatric-specific resources.

### Pediatric nephrology workforce

Overall, the median prevalence of pediatric nephrologists was 0.69 [IQR: 0.03–1.78] per million population (pmp) (Table 2). ISN Eastern and Central Europe (2.41 pmp [IQR: 1.65–2.71]) and North and East Asia (2.33 pmp [IQR: 0.57–4.24]) regions had the highest prevalences of pediatric nephrologists while Africa (0.05 pmp [IQR: 0.00–0.15]) and South Asia (0.07 pmp [IQR: 0.00–0.19]) had the lowest. The prevalence of pediatric nephrologists increased with increasing country income levels; HICs had more than a 50-fold higher prevalence than LICs. More than two-thirds of all countries reported shortages of pediatric nephrologists (n = 116; 69%) with almost all countries in Africa (n = 40; 98%) and LICs (n = 19; 95%) reporting shortages. The calculation of the nephrologist workforce prevalence was limited by the use of absolute country population rather than the age specific populations cared for. The country population data source [6] for the ISN-GKHA was not stratified into age specific groups preventing reporting of pediatric nephrologist and adult nephrologist prevalence per the pediatric and adult populations respectively.

**Table 2:** Pediatric nephrology workforce, shortages, and training program availability, by ISN region and World Bank income group.

	Prevalence of pediatric nephrologists Median [IQR] (pmp)	Perceived shortage of pediatric nephrologists N (%)	Availability of training in pediatric nephrology N (%)		
			Yes	No	Not sure
Overall	0.69 [0.03–1.78]	116 (69)	76 (46)	80 (48)	10 (6)
ISN region					
Africa	0.05 [0.00–0.15]	40 (98)	32 (78)	8 (20)	1 (2)
Eastern and Central Europe	2.41 [1.65–2.71]	10 (63)	3 (19)	12 (75)	1 (6)
Latin America	1.02 [0.69–2.45]	16 (73)	8 (36)	14 (64)	0 (0)
Middle East	1.85 [1.00–2.79]	6 (55)	4 (36)	6 (55)	1 (9)
NIS and Russia	1.68 [1.37–2.72]	6 (60)	2 (20)	8 (80)	0 (0)
North America and the Caribbean	0.00 [0.00–1.31]	7 (58)	10 (83)	2 (17)	0 (0)
North and East Asia	2.33 [0.57–4.24]	4 (67)	0 (0)	6 (100)	0 (0)
Oceania and South East Asia	0.13 [0.00–0.79]	15 (79)	6 (33)	9 (50)	3 (17)
South Asia	0.07 [0.00–0.19]	7 (88)	4 (50)	4 (50)	0 (0)
Western Europe	1.58 [0.96–2.18]	5 (23)	7 (32)	11 (50)	4 (18)
World Bank income group					
Low income	0.03 [0.00–0.06]	19 (95)	16 (80)	4 (20)	0 (0)
Lower-middle income	0.13 [0.07–0.83]	41 (91)	24 (53)	17 (38)	4 (9)
Upper-middle income	1.22 [0.43–1.82]	28 (72)	12 (32)	25 (66)	1 (3)
High income	1.65 [0.71–3.74]	28 (44)	24 (38)	34 (54)	5 (8)

Abbreviations: ISN–International Society of Nephrology; IQR–interquartile range; NIS–Newly Independent States; pmp–per million population

A pediatric nephrology training program was available in 80/166 (48%) countries. Pediatric nephrology training programs were more available in HICs than LICs. Availability of training programs varied across ISN regions and country income groups (Table 2).

### Early identification of CKD in the elderly

The elderly were recognized as a high-risk group in whom early identification of CKD was a routine practice in most responding countries (n = 102; 61%). More than three-quarters of countries in the ISN North and East Asia (n = 5; 83%) and Western Europe (n = 18; 82%) regions routinely tested the elderly for CKD compared to regions where less than half of countries offered such tests to the elderly: Africa (n = 20; 49%), the Middle East (n = 5; 45%), and NIS and Russia (n = 3; 30%) (S6 Table). Similarly, more HICs (n = 47; 75%) than LICs (n = 9; 45%) routinely tested elderly people for CKD.

### Access to kidney failure care and KRT for refugees

**Fig 3:**
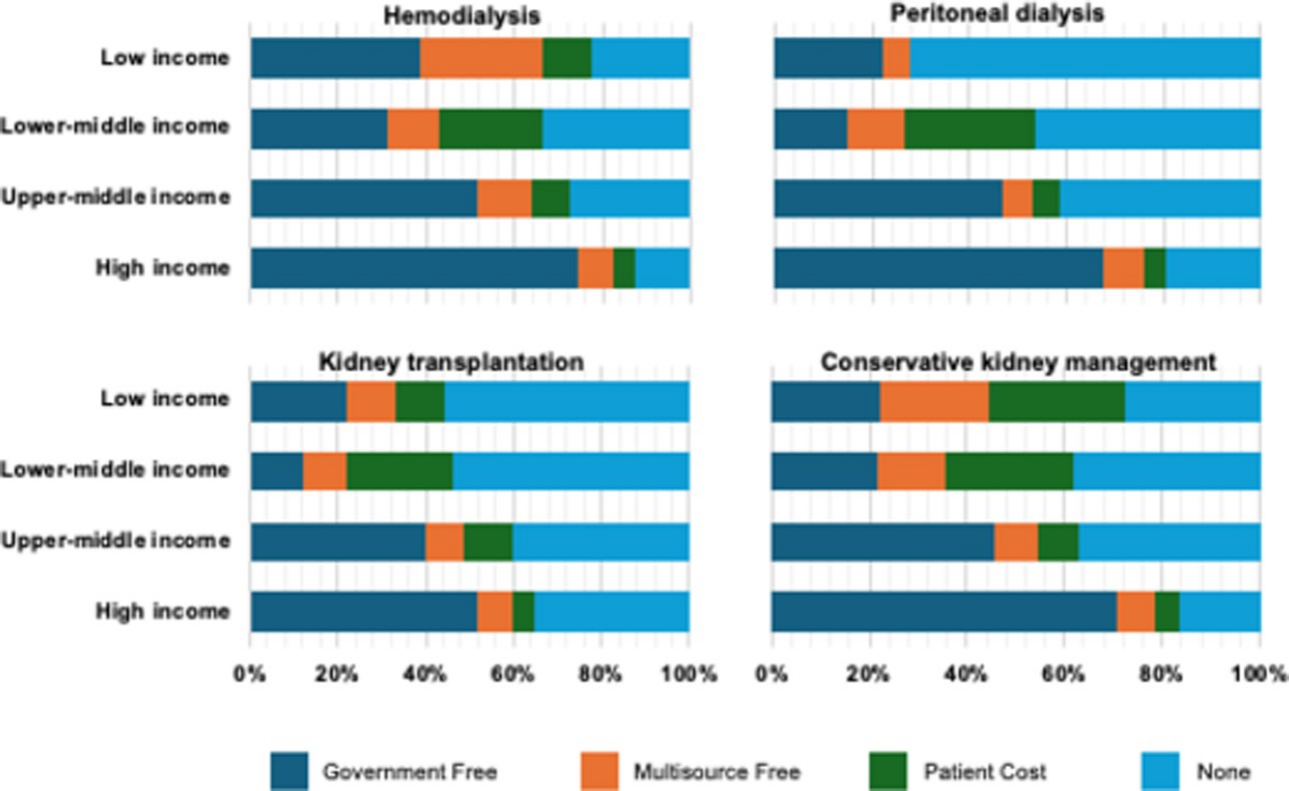
Funding for refugees to access KRT modalities, by World Bank income group*. *Values represent absolute number of countries in each category expressed as a percentage of total number of countries.

Globally, refugees did not have routine access to HD, PD, or KT in 34 (21%), 54 (33%), and 60 (37%) of the 162 countries that were represented in the survey (Fig 3 and S7–S10 Tables). ISN South Asia and OSEA regions had the highest proportions of countries where refugees did not routinely receive HD, PD, KT, or conservative kidney management (CKM). In countries of the ISN Western Europe and Eastern and Central Europe regions, refugees had the highest access to KRT as they were more likely to be covered through public funds free at point of delivery. Variations existed in funding structures for KRT and CKM across ISN regions and country income levels (Fig 3 and S7–S10 Tables).

### Disaster preparedness

Survey respondents were asked about their country’s preparedness for natural disasters. Overall, 52 (32%) countries reported that dialysis facilities had guidelines for providing care in disasters (e.g., earthquake, flood, or drought). While more than half of countries in ISN North and East Asia (n = 5; 83%) and North America and the Caribbean (n = 9; 75%) regions had such guidelines, no countries in NIS and Russia and South Asia reported the availability of such measures (Table 3). Only 14 (9%) countries had a representative on the ISN-RDRTF. Representatives were mainly from the ISN Western Europe (4; 18%), Latin America (5; 24%), Eastern and Central Europe (2; 13%), Africa (2; 5%), and the Middle East (1; 9%) regions, with variations across country income levels (Table 3).

**Table 3:** Availability of guidelines for disaster preparedness and representation on the ISN-RDRTF, by ISN region and World Bank income group (N, %).

	Availability of guidelines for disaster preparedness						Representation in the ISN-RDRTF						
	No		Yes		Unknown		No		Yes		Unknown		Total
	N	%	N	%	N	%	N	%	N	%	N	%	
Overall	81	(50)	52	(32)	29	(18)	106	(65)	14	(9)	42	(26)	162
ISN region													
Africa	29	(74)	4	(10)	6	(15)	30	(77)	2	(5)	7	(18)	39
Eastern and Central Europe	7	(44)	6	(38)	3	(19)	13	(81)	2	(13)	1	(6)	16
Latin America	13	(62)	6	(29)	2	(10)	11	(52)	5	(24)	5	(24)	21
Middle East	5	(45)	5	(45)	1	(9)	8	(73)	1	(9)	2	(18)	11
NIS and Russia	7	(70)	0	(0)	3	(30)	8	(80)	0	(0)	2	(20)	10
North America and the Caribbean	2	(17)	9	(75)	1	(8)	5	(42)	0	(0)	7	(58)	12
North and East Asia	0	(0)	5	(83)	1	(17)	4	(67)	0	(0)	2	(33)	6
Oceania and South East Asia	10	(56)	7	(39)	1	(6)	18	(100)	0	(0)	0	(0)	18
South Asia	5	(71)	0	(0)	2	(29)	5	(71)	0	(0)	2	(29)	7
Western Europe	3	(14)	10	(45)	9	(41)	4	(18)	4	(18)	14	(64)	22
World Bank income group													
Low income	14	(78)	1	(6)	3	(17)	15	(83)	0	(0)	3	(17)	18
Lower-middle income	30	(68)	6	(14)	8	(18)	31	(70)	3	(7)	10	(23)	44
Upper-middle income	23	(62)	7	(19)	7	(19)	29	(78)	4	(11)	4	(11)	37
High income	14	(22)	38	(60)	11	(17)	31	(49)	7	(11)	25	(40)	63

Abbreviations: ISN–International Society of Nephrology; RDRTF–Renal Disaster Relief Task Force; NIS–Newly Independent States

## Discussion

In this analysis, we have demonstrated significant inequities regarding access to kidney care for vulnerable people across all world regions and countries. This is more germane among children, the elderly, and refugees. These groups represent a sizeable proportion of the world population with children constituting almost 30% of the global population and those over 65 years numbering about 10% [7]. Further, there are 29.5 million refugees worldwide, 36% of which are children, and 75% living in LICs and LMICs [8]. The findings highlight the need to have systems in place to identify those who fall into vulnerable groups and learn from studies that explore the challenges in providing care to these individuals. The inequality in pediatric care is particularly notable in our results and is of concern given the recommendations of groups such as the WHO Consortium for the European Review of Social Determinants of Health and the Health Divide that focus on improving social and health care for young families and children as a means of reducing future health risk and vulnerability [9].

The provision of pediatric kidney care is strongly linked to the economic status and political commitment of nations to UHC. It has been well-established that in regions where resources are scarce, children were more disadvantaged than adults in access to healthcare [10]. The lack of insurance and publicly funded healthcare for children, resulting in the high out-of-pocket expenditure expressed in the qualitative responses of this survey, has been reported as an important barrier [11]. In addition to the economic and healthcare priorities of a country, poor awareness about pediatric kidney disease, and lack of robust data from national and international registries are important roadblocks in providing optimal care for pediatric kidney disease [12]. Furthermore, a paucity of pediatric-specific resources, including healthcare personnel, disposables, and equipment, is reported as a common barrier [10]. The shortage of pediatric nephrologists has been reported elsewhere, with the shortages worse in LICs [13]. However, the ISN-GKHA data demonstrate that this perceived shortage is global and substantial, not only in LICs but in HICs as well.

Surveys from the International Pediatric Nephrology Association (IPNA) identified similar barriers to kidney care for children. The lack of trained nephrologists and healthcare personnel, a lack of availability of facilities for KRT, and lack of financial support for healthcare were reported as important challenges in under-resourced regions of the world [14, 15]. Consistently reported barriers to care from the qualitative responses of the current ISN-GKHA emphasize the need for collective and collaborative action between international and regional organizations to address the key roadblocks. Universal healthcare for children, which is supported by public funding, should be a priority. There is a need for governments and international organizations to liaise with industry and to perhaps incentivize production to make pediatric-specific disposables available and accessible globally. While challenges in accessing care for children in low resource settings may relate more to the availability of services and inequities, challenges faced by children in HICs also need to be addressed. For example, one study of Australian Aboriginal and Torres Strait Islander children highlighted systemic and structural challenges that Indigenous children face in comparison to non-Indigenous children while accessing kidney care. These include an increased likelihood to commence HD (rather than PD or a pre-emptive KT), spend much longer on dialysis (22.3 vs. 10.6 months), and if transplanted, an increased likelihood of receiving a deceased donor kidney [16]. It is our opinion, that identifying and recognizing such inequities and biases provides a foundation for addressing these problems and can lead to improved access to and quality of nephrology care for children.

At the other end of the age spectrum, our data demonstrated significant gaps in CKD care and prevention. For instance, less than half of the LICs routinely screen the elderly for CKD, and these countries also have the added risk factors of low socioeconomic status and high comorbidity burden contributing to the CKD risk.

Moreover, the noted disparities in access to kidney care, particularly for KRT, among refugees calls for global attention and concerted action. These gaps in care are wide-spread and systemic as refugees are often described as having reduced access to employment, education, housing, nutrition, and social security programs compared to the general population [17–19]. Often, the unpredictable nature of events causing displacement results in both the refugee and the host countries being unable to access and provide basic services, respectively. The provision of these services is made more complicated among refugees requiring KRT [20–22].

Access to KRT among refugee populations around the world is challenged by multiple socio-economic, political, and specific health related factors. Many countries lacked enabling policies governing optimal refugee health. In particular, understanding the reasons why refugees experience lower access to KRT will facilitate formulation of policies and interventions toward improving access. First, the sudden discontinuation of care and lack of transmission of medical care notes, exacerbated by language barriers, impedes smooth care transition with likely adverse effects on the quality of KRT [23]. Second, the lack of a communicated policy to health workers on how to address refugee care issues is also a factor that hinders access to KRT for refugees. For instance, in one survey, it was reported that even in countries where the government reimbursed KRT, many facilities were unsure of what they were required to do when refugees needed care [24]. Third, given the financial challenges of displaced people, access to KRT may be denied in countries where the cost of KRT is not covered by the government [25, 26]. Unfortunately, as our data show, even in some countries where the government reimburses for the cost of KRT, refugees were excluded from the care the government reimburses. This is a critical global issue that needs addressing particularly with ever increasing cases of natural and man-made disasters displacing more individuals around the world.

It is well-established that disasters, such as earthquakes, floods, and prolonged drought, may be associated with disruptions in power, transportation, communication, water supply, and health services [27]. Patients with kidney disease are particularly vulnerable during disasters due to the need for timely access to advanced medical intervention, availability of specific medications, well-trained personnel, and functioning infrastructure [4]. Pre-disaster plans and preparedness are key to reducing the deaths and morbidity associated with disasters [27–30]. Guidelines at dialysis facilities provide information on steps to take to mitigate the adverse effects of disasters for staff, patients, and their caregivers [4]. Our data showed that disaster preparedness guidelines at dialysis facilities were most commonly available in HICs. Irrespective of country income group, a minority of all countries had dialysis unit disaster response guidelines, reflecting a lack of urgency in preparing for such events.

The ISN-RDRTF was initiated following the 1988 Armenian earthquake when it became apparent that, to prevent kidney failure related deaths, collaborative and coordinated efforts between aid organizations, international and local medical organizations, pharmaceutical companies, dialysis equipment manufacturers, volunteers, donors, and the local ministry of health were required [31–33]. The ISN-RDRTF works to ensure rapid access to dialysis for victims of disaster with acute kidney failure (AKI), and to prevent adverse outcomes among people receiving or needing KRT. Mortality rates amongst those with crush-related AKI undergoing dialysis have improved from about 41% in 1995 to about 15–20% between 1999–2005. This improvement has been achieved with the support of the ISN-RDRTF [30, 34–36]. However, recognized challenges in the ISN-RDRTF carrying out its role include the lack of local expertise and processes for navigating local systems. The ISN-GKHA data demonstrate very few countries with participants on the ISN-RDRTF; perhaps by promoting a model of broader participation on the ISN-RDRTF, guideline development for dialysis units and the ability to carry out disaster-related kidney failure interventions in disaster-impacted countries could be improved.

## Conclusion

While this study has demonstrated significant gaps in access to kidney care for vulnerable people, especially among children, it has highlighted the need for urgent efforts to guarantee equitable access to optimal kidney care for vulnerable populations worldwide. Given ongoing global conflicts and various climate catastrophes that lead to massive displacement of people, there is an increased likelihood that populations that fall within vulnerable categories will continue to increase. This increases the need for developing and implementing policies that ensure adequate access to kidney care for this population. Support for governments, especially in low-resource settings, as well as coordinated international collaborations, will be required to address the inequities. To meet these goals, efforts should span across every level of healthcare from the healthcare provider, patient/family, institution to community with particular focus on patient-centered care, contextual care delivery, and shared decision making for better decisional outcomes [37].

## Supporting information

S1 Table Subset of ISN-GKHA survey questions analyzed. (PDF)

S2 Table Variations in accessing hemodialysis between adults and children, by ISN region and World Bank income group (N, %). (PDF)

S3 Table Variations in accessing peritoneal dialysis between adults and children, by ISN region and World Bank income group (N, %). (PDF)

S4 Table Variations in accessing kidney transplantation between adults and children, by ISN region and World Bank income group (N, %). (PDF)

S5 Table Barriers to equitable kidney failure care and KRT for children. (PDF)

S6 Table Countries that routinely offer early identification of CKD in the elderly population, by ISN region and World Bank income group (N, %). (PDF)

S7 Table Access and hemodialysis funding structures for refugees, by ISN region and World Bank income group (N, %). (PDF)

S8 Table Access and peritoneal dialysis funding structures for refugees, by ISN region and World Bank income group (N, %). (PDF)

S9 Table Access and kidney transplantation funding structures for refugees, by ISN region and World Bank income group (N, %). (PDF)

S10 Table Access and CKM funding structures for refugees, by ISN region and World Bank income group (N, %). (PDF)

S1 Checklist Inclusivity in global research. (DOCX)

S1 Data (XLSX)
