# Supplementary material for: Disparities in kidney care in vulnerable populations: A multinational study from the ISN-GKHA
Source: PLOS Glob Public Health. 2024 Dec 20;4(12):e0004086. doi: 10.1371/journal.pgph.0004086 (PMC11661587; doi:10.1371/journal.pgph.0004086)
Supplement: S1 Table — (PDF) [file pgph.0004086.s001.pdf]

S1 Table. Subset of ISN-GKHA 2023 Survey Questions Analyzed

ISN-GKHA 2023 Question Number	Question	Response Options Analyzed
A.2.1.3	Does organization or delivery of kidney failure (ESKD) care differ between children and adults in your country?	☐ Yes (if possible, please provide brief details)<br>☐ No<br>☐ Unknown
A.2.1.4	Does the access to KRT differ between children and adults in your country?	☐ Yes (if possible, please provide brief details)<br>☐ No<br>☐ Unknown
A.2.2.1	If KRT services are not equal between adults and children, what is the difference in access to hemodialysis (HD)?	☐ More HD access for adults than for children<br>☐ More HD access for children than for adults<br>☐ HD access available for adults, unavailable for children<br>☐ HD access available for children, unavailable for adults
A.2.2.2	If KRT services are not equal between adults and children, what is the difference in access to peritoneal dialysis (PD)?	☐ More PD access for adults than for children<br>☐ More PD access for children than for adults<br>☐ PD access available for adults, unavailable for children<br>☐ PD access available for children, unavailable for adults
A.2.2.3	If KRT services are not equal between adults and children, what is the difference in access to kidney transplant?	☐ More KT access for adults than for children<br>☐ More KT access for children than for adults<br>☐ KT access available for adults, unavailable for children<br>☐ KT access available for children, unavailable for adults
B.2.1.1	Approximately how many nephrologists are there in your country? Please leave blank if unknown.	Pediatric nephrologists:
B.2.2.4	Does a training program for pediatric nephrologists exist in your country?	☐ Yes<br>☐ No<br>☐ Not sure
B.2.2.5	If yes to question B2.2.4 above, what is the length of the training program?	☐ <1 year<br>☐ 1 – 2 years<br>☐ 2 - 4 years<br>☐ >4 years
B.2.3	In your opinion, is there a shortage of any of the following providers in your country for kidney care? Please check all that apply.	☐ **Pediatric nephrologists**
C.18.1	Are there guidelines regarding measures that should be taken for disaster preparedness (i.e. at dialysis facilities) in the event of an earthquake / flood / drought in your country?	☐ Yes (if possible, please provide brief details)<br>☐ No<br>☐ Unknown
C.18.2	Does your country have a representative in the Renal Disaster Relief Task Force?	☐ Yes (if possible, please provide brief details)<br>☐ No<br>☐ Unknown
C.18.3	Are there means of identifying vulnerable populations (i.e. people with housing insecurity, racial/ethnic minorities, people living in poverty, people with food insecurity) in your country?	☐ Yes (if possible, please provide brief details)<br>☐ No<br>☐ Unknown

ISN-GKHA 2023 Question Number	Question	Response Options Analyzed
C.18.4	What best describes your healthcare system funding structure for treatment of CKD and kidney failure (ESKD) in refugee populations?<br><br>-Hemodialysis (some or all aspects of)<br>-Peritoneal Dialysis (some or all aspects of)<br>-Kidney Transplantation (some or all aspects of)<br>=Conservative care (some or all aspects of)	☐ Publicly funded by government and free at the point of delivery<br>☐ Publicly funded by government but with some fees at the point of delivery<br>☐ A mix of publicly funded (whether or not publicly funded component is free at point of delivery) and private systems<br>☐ Solely private and out-of-pocket<br>☐ Solely private through health insurance providers<br>☐ Multiple systems-programs provided by government, NGOs, and communities<br>☐ Other (please specify)<br>☐ N/A (refugees do not routinely have access to treatment for kidney disease)
D.2.1	For which of the following high-risk groups do practitioners in your country routinely offer testing for CKD? (please check all that apply)	☐ The elderly

Abbreviations: ISN-GKHA - International Society of Nephrology-Global Kidney Health Atlas; KRT - kidney replacement therapy; ESKD - End stage kidney disease; CKD - chronic kidney disease
