# Supplementary material for: Disparities in kidney care in vulnerable populations: A multinational study from the ISN-GKHA
Source: PLOS Glob Public Health. 2024 Dec 20;4(12):e0004086. doi: 10.1371/journal.pgph.0004086 (PMC11661587; doi:10.1371/journal.pgph.0004086)
Supplement: S2 Table — (PDF) [file pgph.0004086.s002.pdf]

S2 Table. Variations in accessing hemodialysis between adults and children, by ISN region and World Bank income group (N, %).

	More HD access for adults than for children	More HD access for children than for adults	HD access available for adults, unavailable for children	HD access available for children, unavailable for adults	Total
Overall	46 (74)	4 (6)	12 (19)	0 (0)	62
ISN region:					
Africa	11 (61)	0 (0)	7 (39)	0 (0)	18
Eastern and Central Europe	3 (100)	0 (0)	0 (0)	0 (0)	3
Latin America	8 (67)	2 (17)	2 (17)	0 (0)	12
Middle East	2 (100)	0 (0)	0 (0)	0 (0)	2
NIS and Russia	1 (100)	0 (0)	0 (0)	0 (0)	1
North America and the Caribbean	2 (33)	2 (33)	2 (33)	0 (0)	6
North and East Asia	3 (100)	0 (0)	0 (0)	0 (0)	3
Oceania and South East Asia	7 (88)	0 (0)	1 (13)	0 (0)	8
South Asia	3 (100)	0 (0)	0 (0)	0 (0)	3
Western Europe	6 (100)	0 (0)	0 (0)	0 (0)	6
World Bank income group:					
Low income	6 (67)	0 (0)	3 (33)	0 (0)	9
Lower-middle income	15 (68)	2 (9)	5 (23)	0 (0)	22
Upper-middle income	13 (93)	0 (0)	1 (7)	0 (0)	14
High income	12 (71)	2 (12)	3 (18)	0 (0)	17

Abbreviations: ISN- International Society of Nephrology; HD – hemodialysis; NIS – Newly Independent States
