# Supplementary material for: Disparities in kidney care in vulnerable populations: A multinational study from the ISN-GKHA
Source: PLOS Glob Public Health. 2024 Dec 20;4(12):e0004086. doi: 10.1371/journal.pgph.0004086 (PMC11661587; doi:10.1371/journal.pgph.0004086)
Supplement: S3 Table — (PDF) [file pgph.0004086.s003.pdf]

S3 Table. Variations in accessing peritoneal dialysis between adults and children, by ISN region and World Bank income group (N, %).

	More PD access for adults than for children	More PD access for children than for adults	PD access available for adults, unavailable for children	PD access available for children, unavailable for adults	Total
Overall	28 (53)	18 (34)	3 (6)	4 (8)	53
ISN region:					
Africa	7 (54)	2 (15)	2 (15)	2 (15)	13
Eastern and Central Europe	1 (33)	2 (67)	0 (0)	0 (0)	3
Latin America	6 (60)	4 (40)	0 (0)	0 (0)	10
Middle East	0 (0)	2 (100)	0 (0)	0 (0)	2
NIS and Russia	0 (0)	1 (100)	0 (0)	0 (0)	1
North America and the Caribbean	1 (20)	3 (60)	1 (20)	0 (0)	5
North and East Asia	2 (67)	0 (0)	0 (0)	1 (33)	3
Oceania and South East Asia	6 (75)	1 (13)	0 (0)	1 (13)	8
South Asia	3 (100)	0 (0)	0 (0)	0 (0)	3
Western Europe	2 (40)	3 (60)	0 (0)	0 (0)	5
World Bank income group:					
Low income	3 (60)	0 (0)	1 (20)	1 (20)	5
Lower-middle income	12 (60)	5 (25)	1 (5)	2 (10)	20
Upper-middle income	8 (57)	6 (43)	0 (0)	0 (0)	14
High income	5 (36)	7 (50)	1 (7)	1 (7)	14

Abbreviations: ISN- International Society of Nephrology; PD – peritoneal dialysis; NIS – Newly Independent States
