# Supplementary material for: Disparities in kidney care in vulnerable populations: A multinational study from the ISN-GKHA
Source: PLOS Glob Public Health. 2024 Dec 20;4(12):e0004086. doi: 10.1371/journal.pgph.0004086 (PMC11661587; doi:10.1371/journal.pgph.0004086)
Supplement: S4 Table — (PDF) [file pgph.0004086.s004.pdf]

S4 Table. Variations in accessing kidney transplantation between adults and children, by ISN region and World Bank income group (N, %).

	More KT access for adults than for children	More KT access for children than for adults	KT access available for adults, unavailable for children	KT access available for children, unavailable for adults	Total
Overall	43 (80)	8 (15)	3 (6)	0 (0)	54
ISN region:					
Africa	10 (77)	0 (0)	3 (23)	0 (0)	13
Eastern and Central Europe	2 (100)	0 (0)	0 (0)	0 (0)	2
Latin America	8 (73)	3 (27)	0 (0)	0 (0)	11
Middle East	2 (100)	0 (0)	0 (0)	0 (0)	2
NIS and Russia	2 (100)	0 (0)	0 (0)	0 (0)	2
North America and the Caribbean	3 (60)	2 (40)	0 (0)	0 (0)	5
North and East Asia	2 (100)	0 (0)	0 (0)	0 (0)	2
Oceania and South East Asia	8 (89)	1 (11)	0 (0)	0 (0)	9
South Asia	3 (100)	0 (0)	0 (0)	0 (0)	3
Western Europe	3 (60)	2 (40)	0 (0)	0 (0)	5
World Bank income group:					
Low income	4 (67)	0 (0)	2 (33)	0 (0)	6
Lower-middle income	17 (85)	2 (10)	1 (5)	0 (0)	20
Upper-middle income	10 (83)	2 (17)	0 (0)	0 (0)	12
High income	12 (75)	4 (25)	0 (0)	0 (0)	16

Abbreviations: ISN- International Society of Nephrology; KT – kidney transplantation; NIS – Newly Independent States
