# Supplementary material for: Disparities in kidney care in vulnerable populations: A multinational study from the ISN-GKHA
Source: PLOS Glob Public Health. 2024 Dec 20;4(12):e0004086. doi: 10.1371/journal.pgph.0004086 (PMC11661587; doi:10.1371/journal.pgph.0004086)
Supplement: S5 Table — (PDF) [file pgph.0004086.s005.pdf]

S5 Table. Barriers to equitable kidney failure care and KRT for children.

Factor	Theme	Quotations
Healthcare funding	• Healthcare expenditure<br>• Government funding<br>• Out-of-pocket expenses	• Children are completely covered by the state (Armenia, Uzbekistan)<br>• Social security for children; funds from organizations (Guatemala)<br>• High cost of KF care (Nigeria)<br>• Most privately owned dialysis centers don’t accept children (Turkey)<br>• All children <18 years are paid for by the state (Costa Rica)<br>• Jamaica Kids Foundation provides subsidized care (Jamaica)<br>• High out-of-pocket expenses for children (Papua New Guinea)
Lack of pediatric-specific resources	• Pediatric nephrologists<br>• Pediatric nephrology care/program<br>• Pediatric specific equipment	• There are no pediatric nephrologists; children are not taken care of like adults due to a lack of skill (Burundi)<br>• No pediatric nephrologist, children are followed in adult clinics along with a pediatrician (St Vincent Le Grenadines)<br>• Little or no existence of pediatric nephrology (Fiji)<br>• Pediatric nephrology is less developed than adult nephrology (Congo)<br>• Most dialysis units don’t have consumables for pediatric patients (Ethiopia)<br>• Peritoneal dialysis is not available due to lack of supplies (Malawi)<br>• PD consumables have to be imported (Madagascar)<br>• No medical center that performs transplants in children (Uzbekistan)<br>• No hemodialysis for children (Malawi)<br>• KRT is not available for children (Cape Verde)<br>• Limited resources for children (Iraq)<br>• Dialysis facilities are available for children >12 years (El Salvador)<br>• Priority for children in the deceased donor list (Hong Kong)
Access to KRT	• Distance to care<br>• Centralized services	• Only one children’s hospital in the country (El Salvador)<br>• Kidney failure care for children is available only in two teaching hospitals in the country, where all children are referred (Ghana)<br>• KRT is offered only at tertiary centers (Mexico)<br>• Children from native populations or rural areas cannot access care (Peru)<br>• Difficult transport access from remote areas (Myanmar)<br>• Children who need KRT must go abroad (Aruba)<br>• Caregiver burden, absence from work to travel (Colombia)

Abbreviations: KRT – kidney replacement therapy; KF – kidney failure; PD – peritoneal dialysis
