# Supplementary material for: Disparities in kidney care in vulnerable populations: A multinational study from the ISN-GKHA
Source: PLOS Glob Public Health. 2024 Dec 20;4(12):e0004086. doi: 10.1371/journal.pgph.0004086 (PMC11661587; doi:10.1371/journal.pgph.0004086)
Supplement: S6 Table — (PDF) [file pgph.0004086.s006.pdf]

S6 Table. Countries that routinely offer to screen elderly populations for chronic kidney disease, by ISN region and World Bank income group (N, %).

CKD Screening in the Elderly	N (%)
Overall	102 (61)
**ISN region**	
Africa	20 (49)
Eastern and Central Europe	9 (56)
Latin America	16 (73)
Middle East	5 (45)
NIS and Russia	3 (30)
North America and the Caribbean	7 (58)
North and East Asia	5 (83)
Oceania and South East Asia	14 (74)
South Asia	5 (63)
Western Europe	18 (82)
**World Bank income group**	
Low income	9 (45)
Lower-middle income	25 (56)
Upper-middle income	21 (54)
High income	47 (75)

Abbreviations: ISN - International Society of Nephrology; CKD- chronic kidney disease; NIS – Newly Independent States
