# Supplementary material for: Disparities in kidney care in vulnerable populations: A multinational study from the ISN-GKHA
Source: PLOS Glob Public Health. 2024 Dec 20;4(12):e0004086. doi: 10.1371/journal.pgph.0004086 (PMC11661587; doi:10.1371/journal.pgph.0004086)
Supplement: S7 Table — (PDF) [file pgph.0004086.s007.pdf]

S7 Table. Access and hemodialysis funding structures for refugees, by ISN region and World Bank income group (N, %).

	Publicly funded by government and free at the point of delivery		Publicly funded by government but with some fees at the point of delivery		A mix of publicly funded (whether or not publicly funded component is free at point of delivery) and private systems		Solely private and out-of-pocket		Solely private through health insurance providers		Multiple systems - programs provided by government, NGOs, and communities		Other		N/A (refugees do not routinely have access to treatment for kidney disease)		Total
Overall	63	(39)	17	(10)	12	(7)	12	(7)	1	(1)	18	(11)	5	(3)	34	(21)	162
ISN region:																	
Africa	8	(21)	7	(18)	4	(10)	5	(13)	0	(0)	6	(15)	1	(3)	8	(21)	39
Eastern and Central Europe	13	(81)	2	(13)	0	(0)	0	(0)	0	(0)	0	(0)	0	(0)	1	(6)	16
Latin America	9	(43)	2	(10)	3	(14)	0	(0)	0	(0)	1	(5)	0	(0)	6	(29)	21
Middle East	3	(27)	1	(9)	1	(9)	1	(9)	1	(9)	2	(18)	1	(9)	1	(9)	11
NIS and Russia	2	(20)	2	(20)	2	(20)	1	(10)	0	(0)	1	(10)	0	(0)	2	(20)	10
North America and the Caribbean	1	(8)	1	(8)	1	(8)	2	(17)	0	(0)	1	(8)	2	(17)	4	(33)	12
North and East Asia	1	(17)	1	(17)	1	(17)	0	(0)	0	(0)	3	(50)	0	(0)	0	(0)	6
Oceania and South East Asia	3	(17)	0	(0)	0	(0)	3	(17)	0	(0)	2	(11)	1	(6)	9	(50)	18
South Asia	1	(14)	1	(14)	0	(0)	0	(0)	0	(0)	2	(29)	0	(0)	3	(43)	7
Western Europe	22	(100)	0	(0)	0	(0)	0	(0)	0	(0)	0	(0)	0	(0)	0	(0)	22
World Bank income group:																	
Low income	5	(28)	2	(11)	1	(6)	1	(6)	0	(0)	5	(28)	0	(0)	4	(22)	18
Lower-middle income	6	(14)	6	(14)	5	(11)	8	(18)	0	(0)	5	(11)	1	(2)	13	(30)	44
Upper-middle income	11	(30)	4	(11)	5	(14)	1	(3)	1	(3)	3	(8)	3	(8)	9	(24)	37
High income	41	(65)	5	(8)	1	(2)	2	(3)	0	(0)	5	(8)	1	(2)	8	(13)	63

Abbreviations: ISN - International Society of Nephrology; NGOs – non-governmental organizations; NIS – Newly Independent States
