# Supplementary material for: Disparities in kidney care in vulnerable populations: A multinational study from the ISN-GKHA
Source: PLOS Glob Public Health. 2024 Dec 20;4(12):e0004086. doi: 10.1371/journal.pgph.0004086 (PMC11661587; doi:10.1371/journal.pgph.0004086)
Supplement: S8 Table — (PDF) [file pgph.0004086.s008.pdf]

S8 Table. Access and peritoneal dialysis funding structures for refugees, by ISN region and World Bank income groups (N, %).

	Publicly funded by government and free at the point of delivery	Publicly funded by government but with some fees at the point of delivery	A mix of publicly funded (whether or not publicly funded component is free at point of delivery) and private systems	Solely private and out-of-pocket	Solely private through health insurance providers	Multiple systems - programs provided by government, NGOs, and communities	Other	N/A (refugees do not routinely have access to treatment for kidney disease)	Total
Overall	54 (33)	12 (7)	10 (6)	12 (7)	0 (0)	12 (7)	8 (5)	54 (33)	162
ISN region:									
Africa	4 (10)	4 (10)	3 (8)	4 (10)	0 (0)	3 (8)	5 (13)	16 (41)	39
Eastern and Central Europe	11 (69)	2 (13)	0 (0)	0 (0)	0 (0)	0 (0)	0 (0)	3 (19)	16
Latin America	9 (43)	1 (5)	3 (14)	0 (0)	0 (0)	1 (5)	0 (0)	7 (33)	21
Middle East	2 (18)	1 (9)	1 (9)	3 (27)	0 (0)	1 (9)	1 (9)	2 (18)	11
NIS and Russia	1 (10)	2 (20)	2 (20)	1 (10)	0 (0)	0 (0)	0 (0)	4 (40)	10
North America and the Caribbean	1 (8)	1 (8)	1 (8)	2 (17)	0 (0)	1 (8)	1 (8)	5 (42)	12
North and East Asia	1 (17)	1 (17)	0 (0)	0 (0)	0 (0)	3 (50)	0 (0)	1 (17)	6
Oceania and South East Asia	3 (17)	0 (0)	0 (0)	2 (11)	0 (0)	1 (6)	1 (6)	11 (61)	18
South Asia	0 (0)	0 (0)	0 (0)	0 (0)	0 (0)	2 (29)	0 (0)	5 (71)	7
Western Europe	22 (100)	0 (0)	0 (0)	0 (0)	0 (0)	0 (0)	0 (0)	0 (0)	22
World Bank income group:									
Low income	3 (17)	1 (6)	0 (0)	0 (0)	0 (0)	1 (6)	2 (11)	11 (61)	18
Lower-middle income	2 (5)	3 (7)	5 (11)	9 (20)	0 (0)	5 (11)	3 (7)	17 (39)	44
Upper-middle income	9 (24)	6 (16)	4 (11)	1 (3)	0 (0)	1 (3)	2 (5)	14 (38)	37
High income	40 (63)	2 (3)	1 (2)	2 (3)	0 (0)	5 (8)	1 (2)	12 (19)	63

Abbreviations: ISN - International Society of Nephrology; NGOs – non-governmental organizations; NIS – Newly Independent States
