# Supplementary material for: Disparities in kidney care in vulnerable populations: A multinational study from the ISN-GKHA
Source: PLOS Glob Public Health. 2024 Dec 20;4(12):e0004086. doi: 10.1371/journal.pgph.0004086 (PMC11661587; doi:10.1371/journal.pgph.0004086)
Supplement: S9 Table — (PDF) [file pgph.0004086.s009.pdf]

S9 Table. Access and kidney transplantation funding structures for refugees, by ISN region and World Bank income group (N, %).

	Publicly funded by government and free at the point of delivery		Publicly funded by government but with some fees at the point of delivery		A mix of publicly funded (whether or not publicly funded component is free at point of delivery) and private systems		Solely private and out-of-pocket		Solely private through health insurance providers		Multiple systems - programs provided by government, NGOs, and communities		Other		N/A (refugees do not routinely have access to treatment for kidney disease)		Total
Overall	40	(25)	13	(8)	6	(4)	17	(10)	1	(1)	13	(8)	12	(7)	60	(37)	162
ISN region:																	
Africa	3	(8)	3	(8)	3	(8)	8	(21)	0	(0)	2	(5)	5	(13)	15	(38)	39
Eastern and Central Europe	6	(38)	1	(6)	0	(0)	1	(6)	0	(0)	0	(0)	1	(6)	7	(44)	16
Latin America	7	(33)	2	(10)	2	(10)	0	(0)	0	(0)	1	(5)	1	(5)	8	(38)	21
Middle East	1	(9)	2	(18)	0	(0)	2	(18)	0	(0)	2	(18)	1	(9)	3	(27)	11
NIS and Russia	1	(10)	2	(20)	0	(0)	2	(20)	1	(10)	1	(10)	0	(0)	3	(30)	10
North America and the Caribbean	1	(8)	0	(0)	1	(8)	1	(8)	0	(0)	1	(8)	2	(17)	6	(50)	12
North and East Asia	0	(0)	1	(17)	0	(0)	0	(0)	0	(0)	3	(50)	0	(0)	2	(33)	6
Oceania and South East Asia	2	(11)	0	(0)	0	(0)	3	(17)	0	(0)	1	(6)	1	(6)	11	(61)	18
South Asia	0	(0)	0	(0)	0	(0)	0	(0)	0	(0)	2	(29)	0	(0)	5	(71)	7
Western Europe	19	(86)	2	(9)	0	(0)	0	(0)	0	(0)	0	(0)	1	(5)	0	(0)	22
World Bank income group:																	
Low income	3	(17)	0	(0)	1	(6)	2	(11)	0	(0)	2	(11)	2	(11)	8	(44)	18
Lower-middle income	1	(2)	4	(9)	3	(7)	9	(20)	0	(0)	4	(9)	4	(9)	19	(43)	44
Upper-middle income	8	(22)	6	(16)	1	(3)	4	(11)	1	(3)	2	(5)	1	(3)	14	(38)	37
High income	28	(44)	3	(5)	1	(2)	2	(3)	0	(0)	5	(8)	5	(8)	19	(30)	63

Abbreviations: ISN - International Society of Nephrology; NGOs – non-governmental organizations; NIS – Newly Independent States
