# Supplementary material for: Disparities in kidney care in vulnerable populations: A multinational study from the ISN-GKHA
Source: PLOS Glob Public Health. 2024 Dec 20;4(12):e0004086. doi: 10.1371/journal.pgph.0004086 (PMC11661587; doi:10.1371/journal.pgph.0004086)
Supplement: S10 Table — (PDF) [file pgph.0004086.s010.pdf]

S10 Table. Access and CKM funding structures for refugees, by ISN region and World Bank income group (N, %).

	Publicly funded by government and free at the point of delivery	Publicly funded by government but with some fees at the point of delivery	A mix of publicly funded (whether or not publicly funded component is free at point of delivery) and private systems	Solely private and out-of-pocket	Solely private through health insurance providers	Multiple systems - programs provided by government, NGOs, and communities	Other	N/A (refugees do not routinely have access to treatment for kidney disease)	Total
Overall	49 (30)	22 (14)	8 (5)	18 (1)	0 (0)	17 (10)	9 (6)	39 (24)	162
ISN region:									
Africa	4 (10)	7 (18)	2 (5)	8 (2)	0 (0)	6 (15)	4 (1)	8 (21)	39
Eastern and Central Europe	8 (50)	3 (19)	0 (0)	1 (6)	0 (0)	0 (0)	1 (6)	3 (19)	16
Latin America	9 (43)	3 (14)	3 (14)	0 (0)	0 (0)	1 (5)	0 (0)	5 (24)	21
Middle East	2 (18)	1 (9)	1 (9)	3 (2)	0 (0)	1 (9)	1 (9)	2 (18)	11
NIS and Russia	0 (0)	4 (40)	1 (10)	1 (1)	0 (0)	1 (10)	0 (0)	3 (30)	10
North America and the Caribbean	1 (8)	1 (8)	1 (8)	2 (1)	0 (0)	1 (8)	1 (8)	5 (42)	12
North and East Asia	2 (33)	1 (17)	0 (0)	0 (0)	0 (0)	3 (50)	0 (0)	0 (0)	6
Oceania and South East Asia	2 (11)	1 (6)	0 (0)	3 (1)	0 (0)	3 (17)	1 (6)	8 (44)	18
South Asia	0 (0)	1 (14)	0 (0)	0 (0)	0 (0)	1 (14)	0 (0)	5 (71)	7
Western Europe	21 (95)	0 (0)	0 (0)	0 (0)	0 (0)	0 (0)	1 (5)	0 (0)	22
World Bank income group:									
Low income	1 (6)	3 (17)	1 (6)	3 (1)	0 (0)	4 (22)	2 (1)	4 (22)	18
Lower-middle income	3 (7)	6 (14)	3 (7)	10 (2)	0 (0)	6 (14)	3 (7)	13 (30)	44
Upper-middle income	8 (22)	7 (19)	3 (8)	3 (8)	0 (0)	2 (5)	1 (3)	13 (35)	37
High income	37 (59)	6 (10)	1 (2)	2 (3)	0 (0)	5 (8)	3 (5)	9 (14)	63

Abbreviations: CKM – conservative kidney management; ISN - International Society of Nephrology; NGOs – non-governmental organizations; NIS – Newly Independent States
